# Improved Electrophoretic Deposition of Vertical Single Wall Carbon Nanotubes with Nanoscopic Electrostatic Lenses

**DOI:** 10.3390/mi11030324

**Published:** 2020-03-20

**Authors:** Shanmugamurthy Lakshmanan, Alokik Kanwal, Sheng Liu, Anitha Patlolla, Zafar Iqbal, Somenath Mitra, Gordon A. Thomas, Jeffrey A. Fagan, Reginald C. Farrow

**Affiliations:** 1 Department of Physics, New Jersey Institute of Technology, University Heights, Newark, NJ 07102, USA; sf32@njit.edu (S.L.); alokik@outlook.com (A.K.); shengliu.njit@gmail.com (S.L.); thomasg@njit.edu (G.A.T.); 2 Department of Chemistry and Environment Science, New Jersey Institute of Technology, University Heights, Newark, NJ 07102, USA; ap286@njit.edu (A.P.); zafar.iqbal@njit.edu (Z.I.); somenath.mitra@njit.edu (S.M.); 3 Polymers Division, National Institute of Standards and Technology, 100 Bureau Drive, Gaithersburg, MD 20899, USA; jeffrey.fagan@nist.gov

**Keywords:** carbon nanotubes, nanoprobe arrays, electrophoretic deposition, nanoscopic electrostatic lens, vertical carbon nanotubes, zeta potential, Raman spectroscopy

## Abstract

Under certain conditions, electrophoretic deposition (EPD) of single-wall carbon nanotubes (SWCNTs) onto metal at the base of nanoscale insulating windows can result in a single SWCNT per window, bonded at one end to the metal. During EPD charge, buildup on the insulator creates electrostatic lenses at the windows that control the trajectory of the SWCNTs. The aim is to develop a reproducible process for deposition of individual vertically oriented SWCNTs into each window to enable novel devices. The length of the SWCNTs is shown to be the most critical parameter in achieving results that could be used for devices. In particular, single nanotube deposition in windows by EPD was achieved with SWCNTs with lengths on the order of the window depth. By performing current vs voltage (IV) measurements against a platinum wire in a phosphate buffer and by modeling the data, the presence of the nanotube can be detected, the contact interface can be studied, and the nanotube’s viability for device applications can be determined. These results provide a basis for process integration of vertical SWCNTs using EPD.

## 1. Introduction

There are many applications where a vertical aligned carbon nanotube (CNT) or an array could be employed in an electronic device as the sensing or active device element. Examples include vertical transistors [1,2], biosensors [3,4,5], energy devices [6], and possible combinations of the three. In any of the aforementioned applications, the location and number of CNTs must be controlled with respect to the electrical interconnects. Along with this requirement of precise alignment and registration, there is also a need to control the electronic properties of the CNT such that the device will perform within a specifically defined parameter space. A single-wall carbon nanotube (SWCNT) is required for many device applications. Depositing individual SWCNTs at precise locations remains an engineering challenge for manufacturable applications.

Electrophoretic deposition (EPD) is a process where the particles to be deposited are suspended in a liquid [7,8]. The particles attain a charge due in part to the chemistry of the suspension and can be guided by an electric field. The particles are attracted to and deposited on a substrate that has an opposite charge. In practice, the substrate is generally conducting, such that a potential can be applied relative to a counter electrode to attract the suspended particles. Cathodic EPD refers to positively charged particles deposited on a negative (cathode) substrate. Anodic EPD refers to deposition on a positive substrate. EPD is a versatile technology due to its experimental simplicity, short deposition time, room temperature flexibility, and the ability to deposit a wide variation of materials on arbitrary shapes [7,8].

EPD may offer advantages versus chemical vapor deposition (CVD) [9,10] for depositing CNTs in electronic device configurations [11,12,13]. EPD can provide a potential path for complementary metal oxide semiconductor (CMOS) integration with CNT-based electronics [14]. Another main advantage of EPD is that carbon nanotubes that have been previously sorted for specific electronic properties can be used. Spatial control of the deposition in EPD can be achieved by depositing a thin insulator over metal interconnects and opening windows to the metal at the desired locations using lithography. We have demonstrated key advancements to EPD by using nanoscale patterns, which enables deposition of single SWCNTs at multiple desired locations, oriented vertically, and potentially electrically isolated from other SWCNTs [13]. Together, the spatial and material control opens the door for generating a wide range of devices with predictable behaviors.

Nanoscopic windows used during EPD lead to an electrostatic lensing effect, which enables control of the location and number of deposited nanotubes without the requirement of lithography on the length scale of the diameter of the nanotube [13]. However, (as will be shown) the nanoscale geometry of the windows may also lead to potentially confounding results. Understanding how the SWCNTs interact with these nanoscopic electrostatic lenses is important for achieving acceptable results.

**Figure 1:**
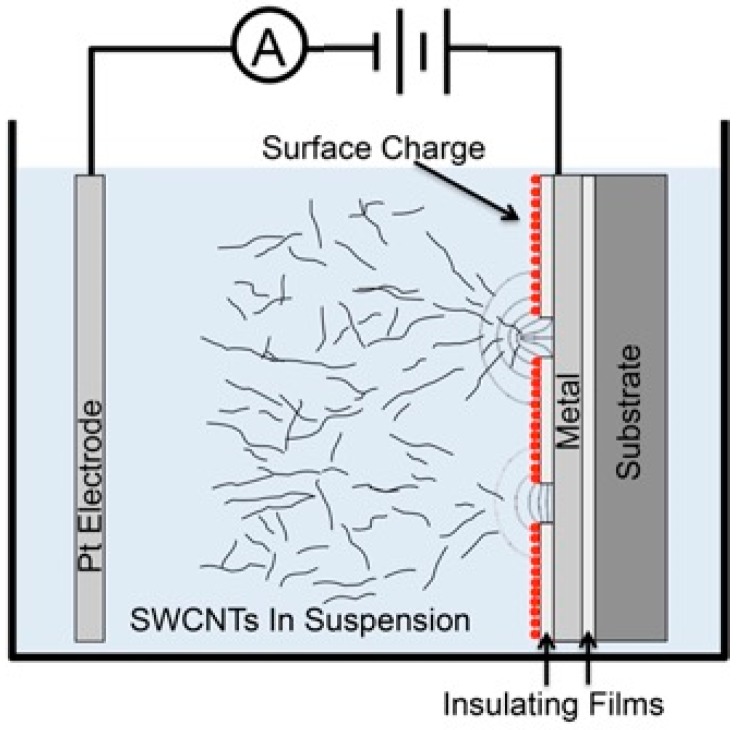
Schematic of electrophoretic deposition (EPD) of carbon nanotubes on metal at the base of nanoscale windows.

The experimental setup is shown schematically in Figure 1. A standard two-electrode configuration was employed during EPD [7,8,11,12,13]. The anodic EPD configuration is shown since the SWCNTs used in this study carried a negative charge in suspension. Initially during deposition, a charge layer was formed on the surface of the insulating layer. This was because of ions in the suspension that are drawn rapidly to the insulator by the applied electric field. Any ions that reach the metal at the base of the window will not produce a surface charge. That is, the potential at the metal surface is assumed to be that which is supplied by the external circuit. The surface charge layer on the insulator creates an electrostatic focusing effect (i.e. nanoscopic electrostatic lens) and directs the nanotube to the window (Figure 1 (bottom window)) [13]. Once a nanotube is deposited inside a window onto the metal (Figure 1 (top window)) and electrical contact is made, the electric field around the window changes, depending on the nature of the nanotube/metal contact. For ideal metal-to-metal contact, the nanotube will acquire the same potential as the metal. A nanotube in the suspension can now be subsequently attracted to the already deposited nanotube. The potential of a semiconducting nanotube after deposition will depend on the Schottky barrier that forms and the sign of the potential during deposition. In any case, if the windows are small enough, the nanotube that deposits first can prevent further nanotube deposition on the metal at the base of the window, but instead drive deposition onto the nanotube that is already connected to the metal.

The degree of verticality of the deposited nanotube will depend on several factors. The initial direction and speed of approach to the window will determine the landing position and angle along with the aspect ratio of the window. The nanotube will attempt to align with the electric field during and after deposition. After the electric field is removed, other forces during rinsing and drying may affect the orientation of the nanotube. The maximum angle away from vertical orientation is limited by the diameter and aspect ratio of the window.

**Figure 2:**
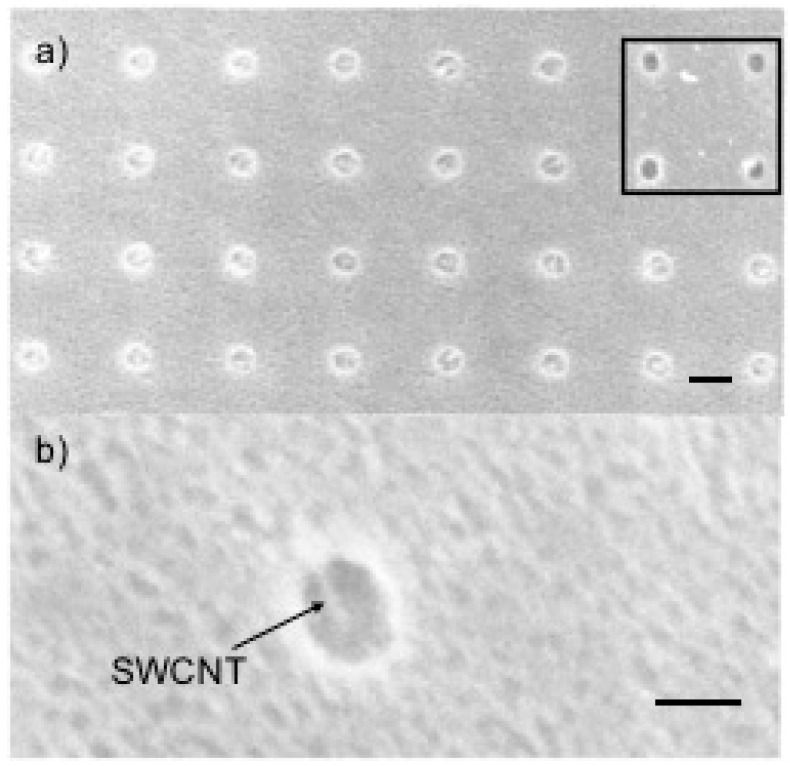
SEM images of SWCNTs deposited in ~100 nm diameter by 50 nm deep vias in SiNx over Co using cathodic EPD. (**a**) Representative area showing that all holes are populated and no SWCNTs are observed on the surface of the SiNx, as compared to before deposition, shown in the inset. Scale bar is 200 nm. (**b**) High magnification image of a via filled with one SWCNT (or bundle) near the center of the via. Scale bar is 100 nm. Reprinted with permission from Goyal et al. [13]. Copyright 2008, American Vacuum Society.

An SEM image of the first reported result is shown in Figure 2 [13]. In this case, the nanotubes were not filtered by type and were given a positive charge in the suspension by adding a magnesium salt, as prescribed by Choi et al [15]. Note that in the surface area around the ~100 nm diameter windows, there are no visible deposited nanotubes and all the windows are filled (see Figure 2a). 

**Figure 3:**
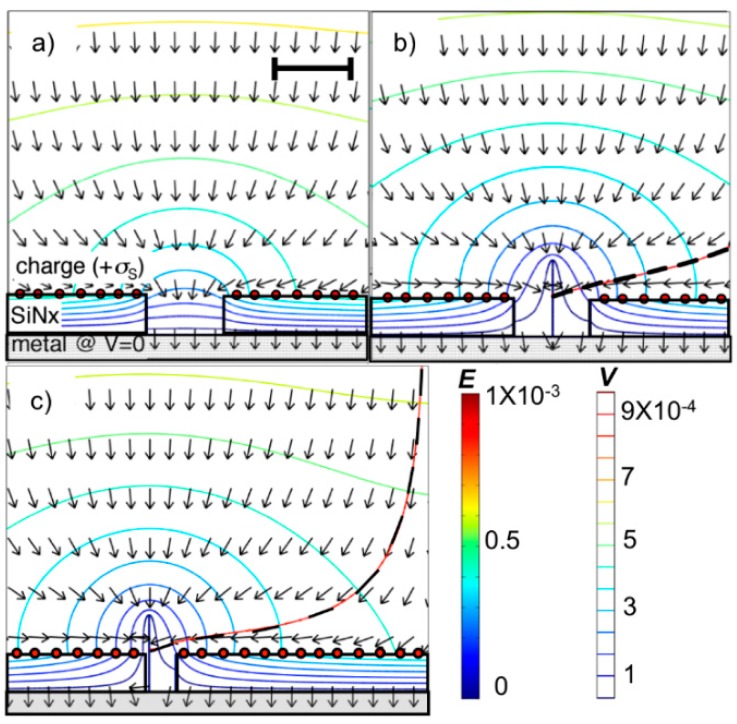
Finite element calculations of electric field for 50 nm deep vias in SiN_x_ over metal with *E* = 10^3^ V/m (contours are equipotential lines and arrows are the electric field): (**a**) 100 nm diameter via and *σ*_S_ = 7.1 × 10^−7^ Coul/m^2^ (annotated with red circles), (**b**) after deposition of 1 nm diameter by 100 nm long metallic nanotube at the center with the trace (dashed line) of a positively charged particle approaching a 350-nm radius from a line that is vertical to the center of the via. (**c**) Electric field and trace of positively charged particle approaching a 40 nm diameter via (*σ*_S_ = 7.1 × 10^−7^ Coul/m^2^) with 1 nm diameter by 100 nm long nanotube attached to the metal at the base of the via. The legend for the electric field and equipotential applies to all figures. The scale bar in (**a**) is 100 nm and represents the magnification of all of the plots. Reprinted with permission from Goyal et al. [13]. Copyright 2008, American Vacuum Society.

The results of a finite element model of the nanoscopic lens effects are shown in Figure 3 [13]. Here, the window geometry and electric field correspond to those used for the results shown in Figure 2. Note that in Figure 3c, even if the nanotube is arbitrarily located to one side of the window, a charged particle in the suspension will not be attracted to the base of a window that is narrow enough.

Induced charge electro-osmosis near the surface of the insulator may contribute to the motion of the nanotubes. In this case, the carriers that drag the liquid have the same sign as the nanotubes and would, therefore, increase the velocity of the nanotubes towards the window. This is particularly the case for nanotubes that are incident away from the center of the window, such as simulated in Figure 3b,c. Since there is no path for the liquid to flow through the window, vortex motion of the liquid due to electro-osmosis is likely close to the window. Therefore, induced charge electro-osmosis may or may not contribute to guiding the nanotube into the windows. It is clear from experiments reported previously [13] and here that there are experimental conditions where (regardless of the contributing effects) SWCNTs can be deposited in nanoscale windows using the EPD method and that the major trends in the experimental results can be largely explained using finite element models that do not take into account fluid motion. The main thrust of this research was to find the experimental conditions for depositing individual SWCNTs in nanoscale windows using EPD.

The experimental results reported here show that there are cases where nanotubes are deposited on the surface near the windows (unlike Figure 2). Those nanotubes that were not connected to the metal at the base of the windows can be rinsed away with varying degrees of success. In general, the EPD process yields variations in results that were identified in the experiments. Many of the applications of this technology are anticipated to require control of the number of deposited SWCNTs in windows and clean surfaces around the windows. A well understood EPD process that leads to uniform and repeatable deposition could pave the way for commercialization of novel vertical SWCNT devices. 

One of the major contributions of this study is the use of SWCNT populations sorted by electrical properties and length for EPD. Previously we used as-synthesized (i.e. unsorted) high-pressure carbon monoxide (HiPCO) nanotubes for our EPD experiments [16]. These SWCNTs contained a mixture of metallic and semiconducting SWCNTs with a wide distribution of lengths and were subject to bundling in suspension [13]. Because these effects led to inconsistent results, it was very difficult to carry out a parametric analysis of EPD with those nanotubes. A key finding, however, was that the long nanotubes were not easily steered to the windows and often fell on the surface of the insulator. It was not clear if the length alone, or a combination of length, surfactant selection, nanotube species, and electric field during EPD, was the root cause of this observation. 

Parametric studies in this contribution were carried out with highly presorted commercial SWCNTs (referred to as Type-I Suspension) that were either 95% metallic or semiconducting and were better protected from bundling by the surfactant choice (although these populations still contained a mixture of lengths). After the primary study was completed, we were also able to obtain a sample of SWNCTs (Type-II Suspension) presorted to contain a distribution of lengths that were much shorter than the Type-I Suspension (average length 83 ± 26 nm), although this population contains a mixture of small diameter semiconducting and metallic SWCNTs. In this paper, we compare SWCNT (both metallic and semiconductor) deposition using Type-I suspension on two kinds of patterns as a function of deposition time. An analysis of the parametric study provides clues to the path for controlling the process to a great extent, which is necessary for commercialization. Finally, we will show that substantially clean EPD of SWCNTs can be obtained when the lengths are on the order of the depth of the windows (i.e. short). Both versions of SWCNTs have been used in a device that performs localized impedance spectroscopy on mobile cells and may lead to a label-free cell detection method [3,4]. The EPD of the short version of the SWCNTs was also used in a nanofabricated discrete enzymatic biofuel cell device that has superior power density performance [6].

One of the challenges of this platform technology is the characterization of the deposited SWCNT. The size of the SWCNT (~1nm diameter) combined with the vertical geometry and the narrow window in which they are deposited (30–50 nm) makes non-destructive electron microscopy difficult. Indeed, just verifying that a SWCNT is deposited is a challenge. The metal to SWCNT interface is also important since it impacts the operation of devices. There has been significant research on the metal to nanotube interface and the role it plays in performance for more conventional planar devices. These devices have their nanotubes lying flat on a substrate and the metal contact connected to the sidewalls of the nanotubes [17,18,19,20]. This information is important to better design devices and to help determine the quality of the fabricated devices during manufacturing. However, there has not been any significant research into the unique contact geometry that results from EPD nanotubes with the use of nanoscopic electrostatic lenses. Vertical geometries have been developed for large area field emission displays, but the physics of the contact interface has not been studied [15,21,22]. Studies of the contacts have been done for carbon nanotubes grown in place vertically [1,2], but not for deposited nanotubes. Electrical characterization of the contacts is difficult in applications where only one end of the SWCNT is connected (such as nanoprobe sensors and fuel cells). Metal contacts could be deposited onto the free ends of the SWCNTs but this would require post processing after SWCNT deposition and would be most useful if the final device requires metal contacts on both ends (i.e. interconnects and transistors).

A method is presented that can non-destructively characterize the deposited nanotubes. The method, which is based on making current vs. voltage (IV) measurements, can rapidly determine the presence of the nanotubes. In addition, this technique has the advantage of probing the contact interface between the nanotube and the metal interconnect. This information will help in understanding the physics of the unique contact geometry and will provide a measure of the health of the device. 

## 2. Materials and Methods 

### 2.1. Device Fabrication and Packaging

**Figure 4:**
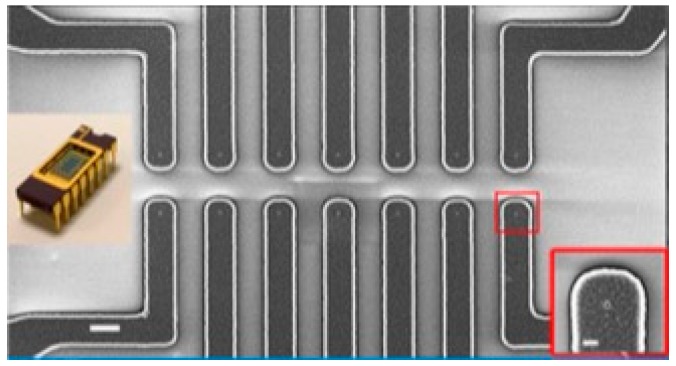
SEM image of the electrodes with the 30–40 nm windows leading down to the metal (1 µm scale bar). Right insert shows a single electrode with the nanoscale window (200 nm scale bar). A packaged device is shown to the left. Reprinted from Kanwal et al. [6] with permission from Elsevier.

Devices for EPD studies were fabricated on 100 mm Si wafers. The device was comprised of metal rails with approximately 30 to 40 nm diameter windows in low-stress silicon nitride (a non-stoichiometric formulation, SiN_x_) over the metal. In one version, there also was a separate pad containing windows with varying window geometry and spacing. Fabrication of the devices started with the deposition of an isolation layer consisting of 100 nm of SiN_x_ and 200 nm of silicon dioxide (SiO_2_) using plasma enhanced CVD (PECVD). A metal stack consisting of 20 nm of chrome, 150 nm of cobalt, and either 50 nm of chrome or titanium was deposited onto patterned resist (suitable for lift-off) using e-beam evaporation. The resist patterning was performed on a GCA Autostep 200 DSW I-line wafer stepper (GCA Corp., Andover, MA, USA). After metal deposition, lift-off was performed to produce a pattern for the metal electrodes with a critical dimension of 1 micron. A low-stress SiN_x_ film (75 nm) was then deposited onto the metal electrodes using PECVD. Contact windows were opened into the nitride using e-beam lithography and reactive ion etching (RIE). After the processing, the wafers were diced and the chips mounted on a chip carrier and wire bonded. After wirebonding, the leads and contact pads were insulated using silicone (MG Chemicals, #4228). Figure 4 shows one version of a completed device ready for EPD. The device shown contains 14 Ti leads that are one micron wide and fan out to contact pads. For EPD experiments, a holder was custom fabricated to isolate liquid from the external electrical leads of the chip carrier.

### 2.2. Preparation of SWCNT Suspensions

Type-I Suspension: Suspensions of purified metallic and semiconducting SWCNTs (95%) were purchased from a commercial vender (NanoIntegris, Boisbriand, QC, Canada). The as-received SWCNTs were placed in an aqueous suspension with a concentration of 0.01 mg/mL. The suspension contains sodium dodecyl sulphate (SDS) and sodium cholate (SC) as surfactants that contribute to the stability of the metallic and semiconducting nanotubes. The surfactant concentration is approximately 1% mass/volume with one part SDS to four parts SC. The general process used by the manufacturer for sorting the nanotubes by diameter and electrical properties is described by Arnold et al. [23]. The diameters of the SWCNTs were specified by the manufacturer to range from 1.2 to 1.7 nm [23]. Raman spectra were recorded from the as-received SWCNTs to verify that they were single wall and sorted into metallic and semiconducting based on well-established spectral features [24]. Our initial electrophoretic deposition trials involved as-received nanotube suspensions. We quickly realized that the nanotubes were too long, causing a large amount of networking to form on the surface of the chips after EPD. Therefore, to shorten the nanotubes, the suspensions were horn sonicated for 6 h (Cole Palmer with a power of 130 Watts and maintained at 75% amplitude). The lengths of nanotubes that were dispersed on the surface of a Si chip were characterized using an SEM (LEO 1530, LEO Electron Microscopy Ltd., Cambridge, UK). The as-received nanotubes ranged from 300 nm to 5 µm in length. After the sonication process, the average length of the metallic nanotubes was 742 nm ± 222 nm and 613 nm ± 273 nm for semiconducting SWCNTs. It is important to note here that it is possible to obtain high-resolution images of SWCNTs that lie on the surface in an SEM, but it is not possible to determine from the images if the SWCNTs are bundled. 

The Type-I suspensions were allowed to settle at least a day before deposition. This was done since, even though the suspensions seemed clear immediately after sonication, some of the nanotubes settled at the bottom of the vials after a day. No further settling was observed after a day. The SWCNTs were taken from a region at the center of the vials that held the suspensions. 

Type-II Suspension: A suspension of SWCNTs was presorted by length using ultracentrifugation [25,26]. The diameters were determined via optical absorption spectroscopy. Based on the observed 40% chirality of (6, 5), the average distribution is about 0.84 nm diameter and semiconducting. To get the true external diameter, the thickness of the excluded volume from the thickness of a sheet of graphene (0.34 nm) is added. Thus, the true external diameter was about 1.2 nm. The nanotubes were in a suspension of 0.15 mg/mL with 1% mass/volume sodium deoxycholate surfactant (SDC) (24 mM) and 6–7% mass/volume iodixanol salt (IS) [27]. The lengths were measured in the SEM as previously described to be 83 ± 26 nm. Before EPD, the SWCNT concentration of the suspension was diluted to ~0.01 mg/mL with the addition of DI water to more closely match that of the Type-I suspension. This reduced the surfactant concentration to 1.6 mM. The diluted suspension maintained its stability throughout the experiments. Table 1 shows a summary of the SWCNT suspensions used in this study.

The zeta potential of each SWCNT sample was measured using a zeta potential analyzer (Zetasizer, Malvern Panalytical, Malvern, UK). The zeta potential is derived from the mobility of the SWCNTs in suspension under the influence of an applied electric field using laser scattering. Specifically, the mobility is calculated from the Doppler shift of the light. The conversion to zeta potential can then be calculated from theory, which will be described in the results. 

### 2.3. EPD Experiment

The experimental set-up for EPD is shown schematically in Figure 1. The chip carrier with the device was mounted on the holder and immersed into the SWCNT suspension. The holder has a platinum electrode mounted 1 cm from the device, which acts as the negative electrode. The device was the positive electrode. The SWCNTs were attracted towards the device when a DC voltage was applied. An ammeter was interfaced to a computer to monitor the current during EPD. 

After the SWCNTs were deposited, the device was immediately rinsed with water followed by isopropyl alcohol and then kept on a hot plate at 70°C for 3 min. The surface of the device was inspected with an optical microscope before SEM diagnostics. If surfactant or any other residue was found, the device was soaked and stirred in hot DI water for a few hours while periodically monitoring the device with an optical microscope until a clean surface was attained. Raman spectroscopy and IV measurements were used to confirm deposition of SWCNTs.

### 2.4. IV Measurement

**Figure 5:**
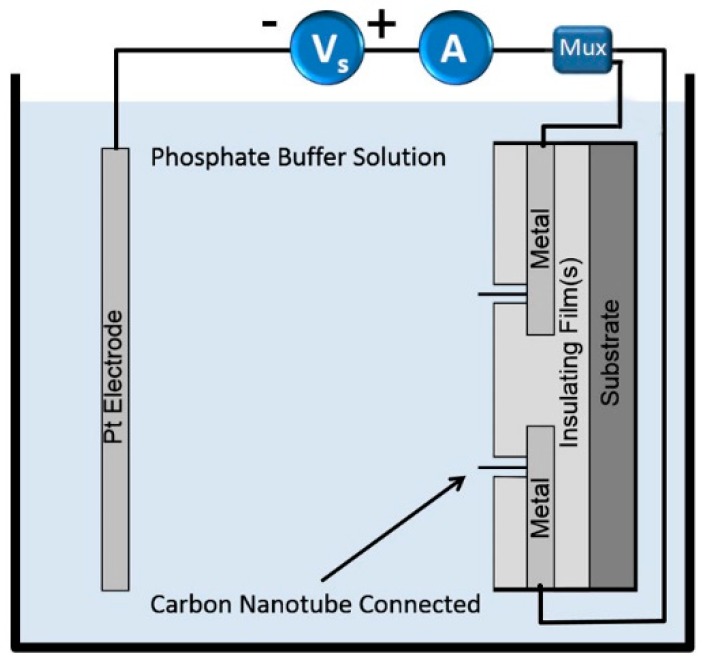
Diagram of experimental setup to measure IV of deposited SWCNTs. A carbon nanotube is selectively connected to the measurement circuit through a multiplexer (Mux).

IV measurements were performed using an HP 4140b picoammeter (Hewlett-Packard, Palo Alto, CA, USA). As shown in Figure 5, chips were placed in a bath of phosphate buffer with one electrode as a platinum wire and the other as the device. Measurements were performed on devices with deposited carbon nanotubes and on devices without nanotubes. The 14 nanoprobe device using the layout shown in Figure 4 was used for the IV experiments. A computer controlled multiplexer circuit was used to switch between windows.

## 3. Results and Discussion

### 3.1. Zeta Potential

For the SWCNT suspensions used in these experiments, the measured zeta potential, *ζ*, ranged from −22 mV to −103 mV. This range suggests that the suspensions were stable. A zeta in the range of *ζ* < −15 mV or *ζ* > 15 mV is considered electrostatically stable [28], which is a prerequisite for EPD [29]. The specific details including the distribution of *ζ* depended on the electronic properties and length of the SWCNTs. An analysis of these contributions is beyond the scope of this paper. The *ζ* were derived from measurements of the electrophoretic mobility, *μ*, which is the rate of migration of the nanotubes in the electric field. It depends on the double layer of charge that forms around the nanotube when in suspension (particularly on the thickness of the double layer compared to the nanotube size). The size, length, and shape of the nanotubes can significantly contribute to the particle motion in EPD. A parameter which compares the particle radius to the Debye length is given by *κa*, where *a* is the radius of the particle and 1/*κ* is the Debye Length [30]. In the case when (*κa* >> 1), the Helmholtz-Smoluchowski (HS) theory for the zeta potential is used. For the surfactant concentrations and SWCNT geometries used in these experiments, *κa* ranges from ~23 to ~323. In the HS limit, the double layer is thin compared to the particle radius. In this case *μ* is given by:

(1) $$\mu =\frac{\varepsilon \zeta }{\eta }$$

where ζ is the zeta potential, *η* is the viscosity, and *ε* is the permittivity of the electrolytic solution [30]. It is important to note the exclusion of polarization effects in this approximation. 

The shape of a particle in suspension also affects its motion under the influence of an electric field. O’Brien et al. [31] showed that the zeta potential decreases for a prolate ellipsoid as the aspect ratio increases. Those results indicate that the HS approximation overestimates the zeta potential by as much as 20% in the high aspect ratio limit (such as for SWCNTs). This overestimate of *ζ* will still allow us to use the HS theory to narrow the possible explanations of the trends in the EPD results. The *ζ* results for the SWCNTs using the HS theory are listed in Table 2. 

It is difficult to make specific conclusions about the relative *ζ* values of the table since many factors can influence the measurement, including the electronic properties of the SWCNTs, the geometry (particularly length), and the surfactant (including concentration) [28]. The result for the short nanotubes (Type-II) has three values that may be attributed to a mixture of metallic and semiconducting SWCNTs and possibly an impurity in the suspension. If the concentration of surfactants is above the critical micelle concentration (CMC), it may affect the zeta potential results. In a suspension of CNTs, the concentration of free surfactant may increase until the CMC, where aggregates of surfactant can form. These aggregates are detectable in light scattering experiments (such as the zeta potential measurement). Aggregates of surfactant may have affected the short nanotube zeta potential results since there are multiple peaks, but only if there were aggregates of surfactant remaining after dilution of the samples with DI water. In the final suspension, the concentration of sodium deoxycholate is less than CMC, which is ~2.6 mM for small aggregate formation and ~6.7 mM for stable micelle aggregation with an aggregation number of ~3.2 (all values at room temperature).

The main conclusion that can be drawn from the zeta potential experimental results is that all of the measured *ζ* values were within a range that would indicate a stable suspension. It is also important to note that the *ζ* values for the short SWCNTs indicate that their effective charge is comparable but more negative than the longer SWCNTs. This may be attributed to differences in the surfactants used in the suspensions. This is significant since under the same deposition conditions with a more negative *ζ***,** the shorter nanotubes in these experiments will reach a higher speed under the influence of the applied electric field.

### 3.2. Electrophoretic Deposition

**Figure 6:**
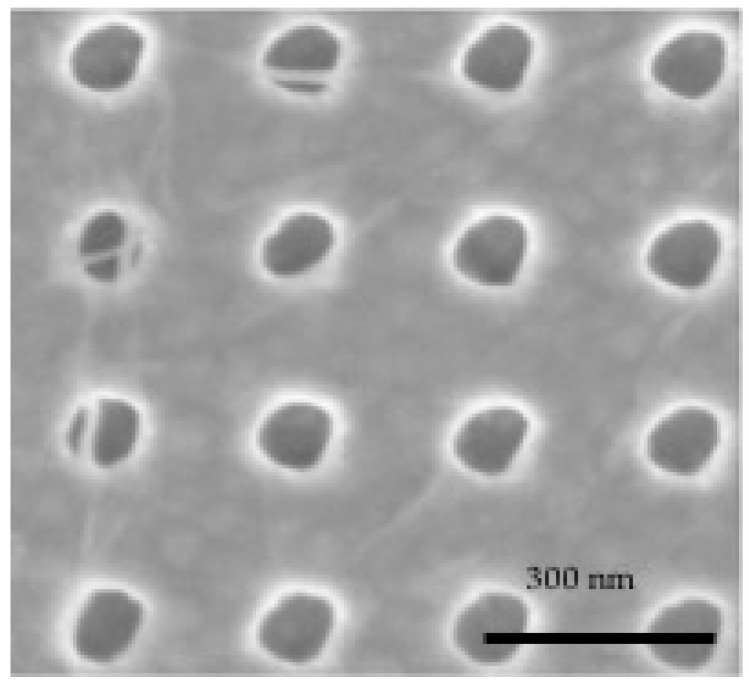
SEM images of EPD results for 2 s deposition times using 10 V for s-SWCNTs (semiconducting).

**Figure 7:**
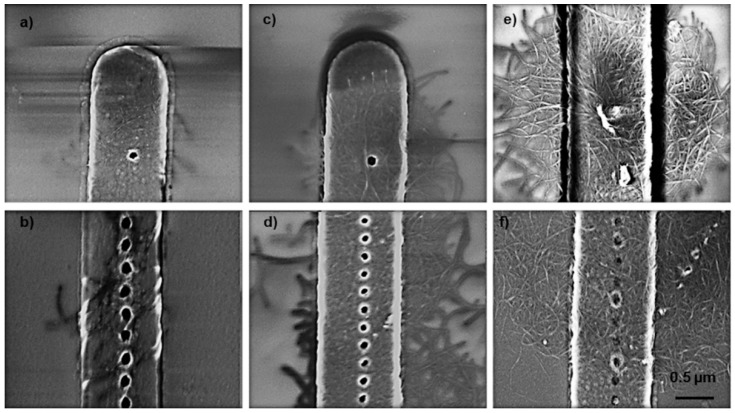
SEM images of EPD results with m-SWCNT (average length 781 nm) with deposition times of (**a**) and (**b**) 1 s, (**c**) and (**d**) 2.5 s, and (**e**) and (**f**) 5 s in isolated windows (**a**,**c**,**e**) and linear dense arrays (**b**,**d**,**f**).

The deposition results for Type-I nanotubes were compared as a function of time. There was a noticeable variation between semiconducting and metallic SWCNTs (s-SWCNT and m-SWCNT) during these experiments. The key difference was that the deposition rate of the s-SWCNTs during EPD was less than that for the m-SWCNTs. This could be expected since the **ζ** of the m-SWCNTs was more than twice that of the s-SWCNTs. Figure 6 shows a representative example result for the s-SWCNTs during a deposition of 2 s. Note that there are SWCNTs deposited across the windows and on the surface. Except for the difference in deposition rate, the EPD for the semiconducting and metallic types were similar. We focus here on the m-SWCNT results to demonstrate EPD of longer nanotubes in nanoscale windows. The deposition time was varied from 1 s to 5 s for two kinds of patterns: isolated windows ≅ 40 nm diameter, and linear dense arrays ≅ 50 nm diameter with 150 nm spacing from center to center. Representative results are shown in Figure 7 for the isolated window pattern and the linear dense arrays. The Type-I results are for a 10 V potential (*E* = 10^3^ V/m). Changing the potential modified the pattern of deposited SWCNTs but did not result in single nanotube deposition in the windows.

As the deposition time increased in steps from 1 s to 5 s, the area of networked m-SWCNTs increased around the isolated windows (see Figure 7a, c, and e). That is, in this range of time, the radius of the networked nanotubes increases over time. Beyond 5 seconds of deposition, the radius of networked nanotubes increases at a slower rate, indicating that a saturation effect might occur for longer times. From the sequence of results, it appears that a 1 second deposition time is too long to achieve results for the Type-I suspension, where a single nanotube is deposited in the window and the surface outside the window is free of deposited nanotubes. The distribution of deposited nanotubes is a function of the electrodynamics of the nanotubes during EPD and particularly their length. Correspondingly, the electric field distribution changes around the window once nanotubes are deposited and this field then varies as a function of the length of nanotubes in the suspension. Of course, the final distribution of nanotubes also depends on the rinsing steps used after deposition. Similar results were observed when the electric field was left on during rinsing and drying. However, surface tension of the water during drying may have an effect on the structure of the networks that could not be accounted for in these experiments since critical point drying was not employed. It is important to note that the results shown in Figure 6 and Figure 7 would not be suitable for most of the anticipated device applications for vertically oriented SWCNTs.

**Figure 8:**
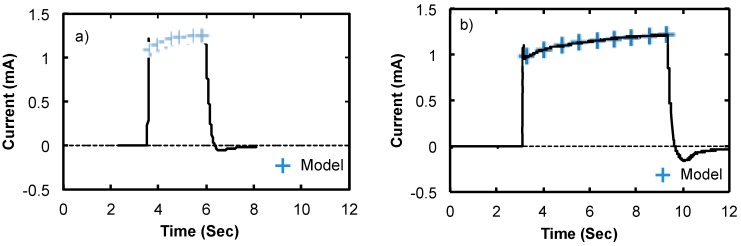
Plots of current vs. time during EPD of long m-SWCNTs for an isolated window (**a**) and a dense linear array (**b**).

The current during EPD for the cases shown in Figure 7c,f is plotted in Figure 8. These are typical results for the Type-I suspension. There is a slight peak when the power is switched on that was determined to be an artifact of the electronics setup. Once deposition was underway, there is an almost linear increase in current early in the deposition that starts to level off after a few seconds. The time dependence of the current, I(t), was modeled using variation of the Hill Equation [32]:

(2) $$\frac{1}{I\left(t\right)-m_{0}}=\frac{1}{m_{1}t}+\frac{1}{m_{2}}$$

where *m_i_* are constants. At short times, the current rises at the rate *m_1_*, which for Figure 8a,b are 0.229 mA/sec and 0.118 mA/sec, respectively. Extrapolating beyond the actual deposition times predicts that the current saturates at *m*_0_ + *m*_2_, which are 1.341 mA and 1.335 mA for the results in Figure 8a,b, respectively. Since the current is through the liquid suspension, it is related to the reaction of ions with the electrodes (i.e. the sample and the Pt wire). The specific reactants and their kinetics are not known for these experiments and there may also be affects at the electrodes due to hydrolysis of water because of the potentials that were used in some cases. With any combination of reactions at the electrodes, the current will be proportional to the area of electrodes available to the reactants. Due to the electric field, once a SWCNT makes contact with the metal at the base of a window, all of the current will flow through the nanotube to the metal. It is conceivable that the time dependence of the current during EPD reflects the change in effective area of SWCNTs that are reacting with the ions in the liquid. It is important to note that it is not possible to visually monitor the SWCNTs during EPD, but the SEM results and current measurements are consistent with the interpretation of SWCNT connected area increasing over time with the Type-I suspension.

**Figure 9:**
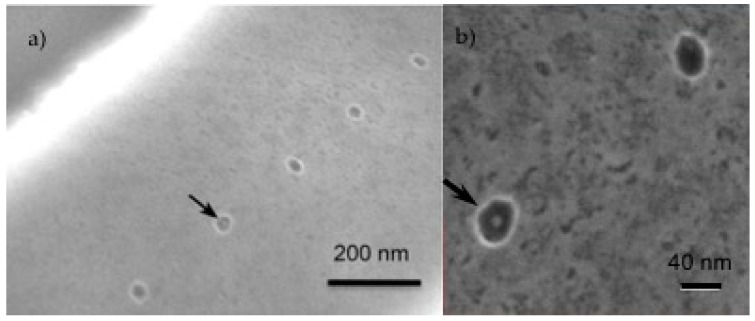
(**a**) SEM image of EPD result with SWCNTs sorted to have an average length 83 ± 26 nm. Higher magnification image (**b**) is reprinted from Kanwal et al. [6] with permission from Elsevier. The deposition time was 10 min at 2.5 V.

A reasonable hypothesis is that, if the length of the SWCNT is small enough, there is a high probability that the SWCNT that is closest to the window when the electric field is turned on will drift into the window and deposit on the metal. This would change the dynamics of the deposition for short SWCNTs and lead to less deposition on the surface of the insulator because most will be directed even further away from the surface by the electric field of the SWCNT deposited in the window, combined with those that have connected to it. It may be possible to have a condition where rinsing will be more effective at leaving a single SWCNT in the window. The example shown in Figure 9 was from a device that was subsequently fabricated into a single-planar biofuel cell with only 2 micron spacing between the anode and cathode [6]. Networking of nanotubes in the manner shown in Figure 7 would have caused the biofuel cell to fail because of shorting of the anode and cathode. There are interdigitated rails of ~40 nm windows spaced on 200 nm centers. SWCNTs that were sorted to have an average length 83 ± 26 nm (e.g. Type-II SWCNTs) were deposited in the windows. The electric field (E = 2.5 × 10^2^ V/m) was turned off during rinsing and drying. The results for a long deposition time as shown in Figure 9 are dramatically different when compared to the longer SWCNTs (see Figure 7). The surface around the windows is clean (i.e. no SWCNTs) (see Figure 9a). The SWCNTs deposited in the windows are visible at higher magnification as shown in Figure 9b and in previously reported higher magnification SEM images where the short SWCNTs appear to be deposited in the windows and oriented vertically [3,6].

**Figure 10:**
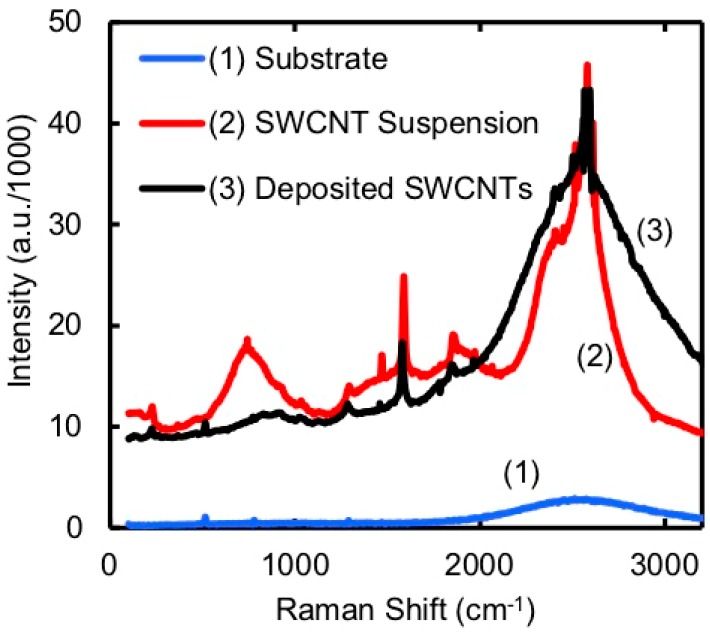
Raman spectra taken of nanotubes in suspension, and the before and after deposition of nanotubes on the rail structure. The Raman lines associated with nanotubes appear in the suspension and only on the device after deposition, indicating the presence of nanotubes.

The short SWCNT deposition was also verified by performing micro-Raman spectroscopy [24] in the area where the SWCNTs were deposited and the surface was otherwise clean. The spectra shown in Figure 10 recorded from the EPD sample matched those taken from the SWCNT suspension and contained well-known peaks characteristic of SWCNTs [24]. The D band (~1350 cm^−1^), G-band (1550–1605 cm^−1^), and G’-band (~2700 cm^−1^) are clearly resolved. The broad peak just below 1000 cm^−1^ is not characteristic of SWCNTs but does not appear in the spectrum of deposited SWCNTs. Otherwise, the spectrum of the deposited SWCNTs is a convolution of the substrate and the suspension. 

**Figure 11:**
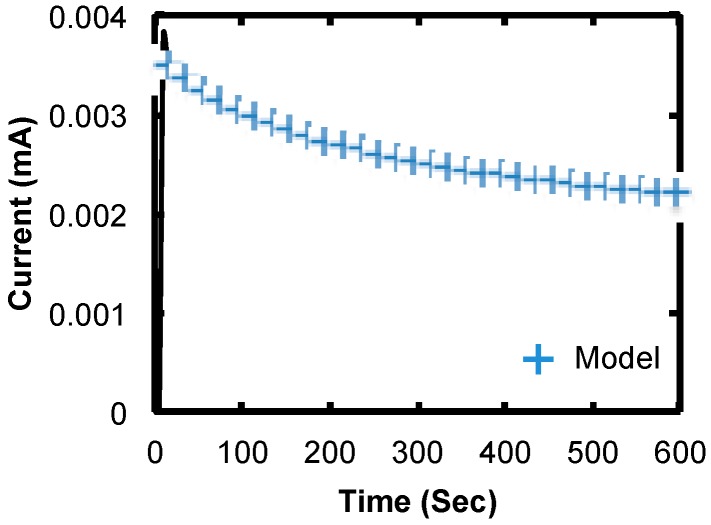
Plot of current vs. time during EPD for short SWCNTs from a dense linear array of windows.

The current recorded during EPD of the short SWCNTs shown in Figure 11 indicates that the dynamics are very different than for long SWCNTs. After an initial rise in the current, there is an almost linear decrease that starts to level off after some time. The behavior also fits the model given in Equation (2), but with the opposite trend of currents recorded for EPD of long SWCNTs. In the plot in Figure 11 early in the deposition, the current decreases at the rate *m*_1_ = −7.8026 µA/Sec. In this case, extrapolating beyond the actual deposition times predicts that the current saturates at *m*_0_ + *m*_2 =_ 0.00162 mA. This decreasing current during EPD is consistent with SWCNTs forming networks above the surface over time rather than depositing on the surface. In this case, it is conceivable that the SWCNTs deposit farther away from the surface over time and that the electric field will direct the charged reactants in the suspension farther away from the surface to react with those SWCNTs. Since the EPD process is random, the structure of the deposited SWCNTs may cause some screening of SWCNTs in closely spaced windows and reduce the effective surface area that is available to reactants and thereby reduce the current over time. It is important to note that the magnitude of the current for the short versus long nanotubes is largely attributed to the difference in patterned area and not to the dynamics of the deposition.

### 3.3. IV Measurements

**Figure 12:**
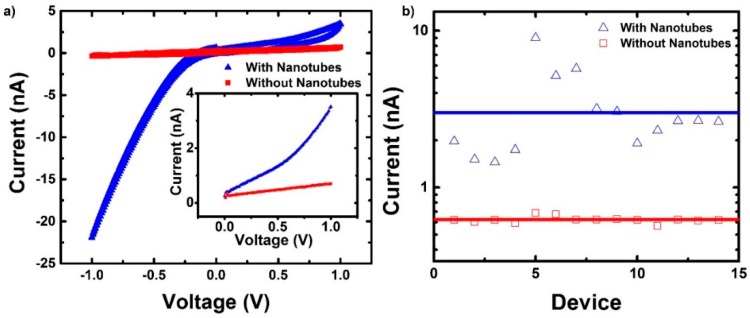
**(a**) The average current vs. voltage curve of devices with and without nanotubes. The scan goes from 0 V to 1 V then down to −1 V and finally back to 0 V. Insert shows the positive range of the voltage from 0 to 1 V. (**b**) The range of currents for 14 devices, with and without nanotubes.

Figure 12 shows the results of IV measurements recorded on samples after Type-II (short) SWCNT deposition compared to samples before deposition. The single-window layout devices were used (see Figure 4) and the SWCNT EPD conditions were the same that produced the result shown in Figure 9. That is, a 10 min deposition was performed using a potential of 2.5 V (*E* = 2.5 × 10^2^ V/m).

During the IV sweep, the voltage starts at 0 V and is varied up to 1 V. Next, the voltage is scanned down to −1 V, and finally brought back up to 0 V. The fact that one electrode is a carbon nanotube connected to a Ti lead and the counter electrode is a macroscopic Pt wire accounts for the asymmetry in the IV curve for these devices. The difference in electrode geometries leads to differences in reaction at the electrode liquid (phosphate buffer) interface. If the voltage is positive, electrons enter the Pt and are used for electrochemical reactions with positive ions at the Pt buffer interface. Negative ions move to the carbon nanotube and through electrochemical reactions, electrons are transferred to the nanotube and are travel into the Ti, through the Ti/carbon nanotube interface. A Schottky barrier for holes is present between the Ti metal and the carbon nanotube. The nanoscale window geometry combined with the geometry of carbon nanotube confines the electrochemical reactions to the exposed tip of the carbon nanotube, where the induced electric field is the largest. When the voltage is negative, electrons travel towards the nanotube though the Ti and are used for electrochemical reactions with the phosphate buffer. Unlike in the positive voltage, the higher current in the IV curve suggests that there is no significant barrier for electron or hole conduction in either electrode.

Measurements on samples without nanotubes resulted in a lack of asymmetry in the IV curves. In addition, the IV curves were in the range of hundreds of picoamps, as opposed to nanoamps for devices with nanotubes. The lack of asymmetry and lower current when no nanotubes were deposited suggest the role that the nanotubes play. One possible explanation is that the nanotube allows for easier access to the buffer. Without a nanotube, the buffer has to penetrate a 30–40 nm window that is 75 nm deep, which is exceptionally difficult given the small geometry, the aspect ratio, and the surface tension of water. When a nanotube is connected to a device, it may extend beyond the hole and into the liquid, allowing for intimate contact between the buffer and the nanotube. Considering the unique size and aspect ratio of the windows and the length of the nanotubes (mean length ~83 nm), we can infer that nanotubes are relatively vertical. With a window diameter of 30–40 nm and a depth of 75 nm, the maximum angle from completely vertical is 21 to 28 degrees. This would allow for the nanotube to just reach the surface of the chip. The one to two order of magnitude difference in the current between devices with and without nanotubes could be also attributed to the differences in aspect ratio. The sharper tips of the 1.2 nm diameter nanotubes compared to the 30–40 nm diameter windows enhance the electric field. The asymmetry in the IV curve can be an indication of the presence of a nanotube within the device.

The effects of the surfactant used during deposition of the SWCNTs also need to be considered. A stable suspension is critical to the EPD process. This involves mixing a surfactant with the SWCNTs in an aqueous solution. The surfactants wrap around the SWCNTs [25]. After deposition, the surfactant rinses clean from the substrate, but it is difficult to detect the extent that it rinses from the SWCNT. The surfactant may act as an effective passivation of the sides of the SWCNTs and further concentrate the charge conduction to the tip.

Further evidence is provided by looking at the distribution of current ranges across the 14 devices. For the case with nanotubes, the variation in the range of currents is much larger than that of devices without nanotubes, as shown in Figure 12b. Devices without nanotubes showed a very narrow distribution, staying in the range of 10^−10^ A, while devices with nanotubes show a much larger distribution ranging from 10^−9^ to 10^−8^ A. The large variation suggests the conduction in the circuit is strongly coupled to the presence of the carbon nanotube. The distribution is also an indication of variation in the devices. This variation could be due to some devices having single nanotubes, while others could have small bundles. This would change the contact area, giving the variation of current range. Another possible reason for the large variation in current could be the contact interface between the nanotube and titanium. 

**Figure 13:**
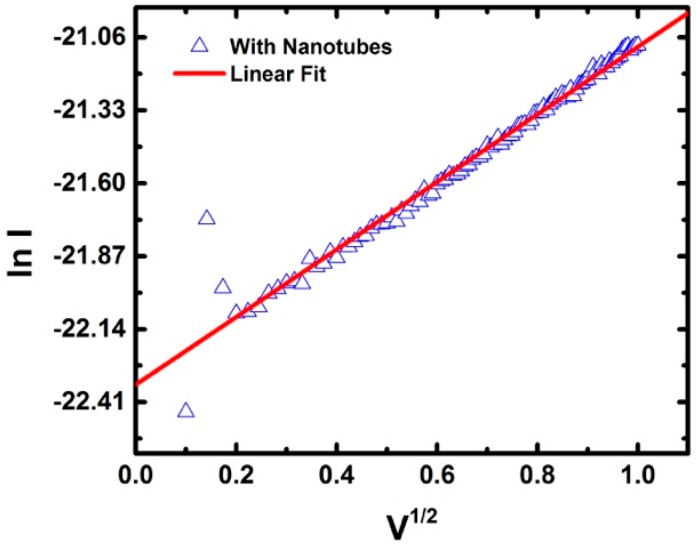
Fit for a Schottky barrier model for the average IV curve, from 0 to 1V.

To look at the contact interface, the IV curves from 0–1 V (Figure 12a insert) were fitted with a Schottky barrier (SB) model [33]. The expression for the current, *I,* is:

(3) $$I=AA^{*}T^{2}\exp\left(-\frac{\phi_{b}}{kT}\right)\exp\left[\frac{e}{kT}{\left(\frac{eV}{4\pi \varepsilon_{0}\varepsilon_{r}}\right)}^{\frac{1}{2}}\right]$$

where *A* is the contact area, A* is the Richardson constant, *T* is temperature in kelvin, $\phi_{b}$ is the barrier height, *k* is Boltzmann constant, *e* is the fundamental charge, *V* is the applied voltage, ε_0_ is the permittivity of free space, and ε_r_ is the dielectric constant of a nanotube. The resulting fit of the average current is shown in Figure 13. Using a diameter of 1.2 nm, the Schottky barrier height for the average IV (0–1 V) curve is 0.15 eV ± 0.02 eV. The 0–1 V range was chosen since the system was unmodified by a previous voltage. After 1V, a hysteresis was observed, indicating that the interface between the contact and the buffer was changed, either by charge storage through a double layer or another electrochemical reaction. The calculated barrier heights derived from the measurements reported here agree with SWCNT devices with titanium contacts [20]. This analysis makes an assumption about the contact area that has not been verified. However, the evidence of a metal-semiconductor interface is compelling.

## 4. Conclusions

The EPD results indicate that, of all the factors that affect the deposition of SWCNTs in nanoscale windows, the length of the nanotubes is critical to a result that may be used in device fabrication. For the longer SWCNTs that were deposited using EPD, there was no experimental condition that led to a suitable control over the number deposited. This included low voltage deposition, where hydrolysis was not a factor. There was rather a consistent tendency to form networks of SWCNTs around the windows. For the majority of device applications, this would not be suitable. The time-dependent EPD experiments imply that for the m-SWCNTs, the deposition time would have to be less than a second to get only a single nanotube deposited and that a fraction will not reach the metal at the base of the window. Again, this would not lead to an acceptable solution for largescale integration.

The situation is very different if the SWCNTs are shorter with lengths on the order of the depth of the windows that were used in these experiments. In the case of EPD with shorter SWCNTs, the results indicate that the deposition can be self-limiting of the number deposited. This was clearly evident in the preliminary results where the deposition time was 10 min. It can be argued that only one SWCNT will deposit if they are short enough to be captured by the window and that the first approaching SWCNT successfully makes contact with the metal at the base of the window. Based on these results, it is conceivable that with further process development, a large-scale integration strategy for vertical devices using pre-sorted SWCNTs deposited by EPD can be achieved. Large-scale integration will require more parametric studies and a comprehensive finite element model that takes into account all factors, including (but not limited to) the geometry and pattern density of the windows, along with the length of the SWCNTs.

The nanoscale geometry and vertical orientation of the SWCNTs devices, although potentially useful, offer significant obstacles for physical diagnostics. An IV measurement of the SWCNT device in a phosphate buffer solution with a platinum counter electrode has been demonstrated to detect when a semiconducting SWCNT is deposited and making an electrical contact with the metal.

It is important to point out that both the deposition and IV methods that are presented involve electrochemical interactions with electrodes. It is reasonable to suggest that follow-up studies of both the EPD and IV effects be performed using a three-electrode configuration with a reference electrode. This may be particularly useful in understanding the nature of the SWCNT/metal contact.

## Figures and Tables

**Table 1:** SWCNT suspensions.

SWCNT Type	Diameter (nm)	Length (nm)	SWCNT (mg/mL)	Suspension
Type-I (m-SWCNT)	1.2–1.7	742 ± 222	0.01	1% mass/volume with one part SDS to four parts SC
Type-I (s-SWCNT)	1.2–1.7	613 ± 273	0.01	1% mass/volume with one part SDS to four parts SC
Type-II (Short)	1.2	83 ± 26	0.01	0.07% mass/volume SDC and 0.4–0.47% mass/volume IS

**Table 2:** Zeta potential of SWCNT suspensions.

SWCNT Type	*ζ* (mV)	FWHM (mV)
m-SWCNT	−50	19
s-SWCNT	−19	16
Short (1)	−103	10
Short (2)	−72	9
Short (3)	−46	20

## References

[1] Franklin A.D., Sayer R.A., Sands T.D., Janes D.B., Fisher T.S. (2009). Vertical carbon nanotube devices with nanoscale lengths controlled without lithography. IEEE Trans. Nanotechnol..

[2] Graham A.P., Duesberg G.S., Hoenlein W., Kreupl F., Liebau M., Martin R., Rajasekharan B., Pamler W., Seidel R., Steinhoegl W. (2005). How do carbon nanotubes fit into the semiconductor roadmap?. Appl. Phys. A Mater. Sci. Process..

[3] Kanwal A., Lakshmanan S., Bendiganavale A., Bot C.T., Patlolla A., Raj R., Prodan C., Iqbal Z., Thomas G.A., Farrow R.C. (2013). Scalable nano-bioprobes with sub-cellular resolution for cell detection. Biosens. Bioelectron..

[4] Thomas G.A., Prodan C., Farrow R., Kanwal A., Bayconi N., Bendiganavale A. (2013). Dynamic, nano-probe measurement of complex impedance near single yeast cells. Biophys. J..

[5] Claussen J.C., Franklin A.D., ul Haque A., Porterfield D.M., Fisher T.S. (2009). Electrochemical biosensor of nanocube-augmented carbon nanotube networks. ACS Nano.

[6] Kanwal A., Wang S.C., Ying Y., Cohen R., Lakshmanan S., Patlolla A., Iqbal Z., Thomas G.A., Farrow R.C. (2014). Substantial power density from a discrete nano-scalable biofuel cell. Electrochem. Commun..

[7] Amrollahi P., Krasinski J.S., Vaidyanathan R., Tayebi L., Vashaee D., Aliofkhazraei M., Makhlouf A.S.H. (2016). Electrophoretic deposition (EPD): Fundamentals and applications from nano- to microscale structures. Handbook of Nanoelectrochemistry: Electrochemical Synthesis Methods, Properties, and Characterization Techniques.

[8] Besra L., Liu M. (2007). A review on fundamentals and applications of electrophoretic deposition (EPD). Progress Mater. Sci..

[9] Maschmann M.R., Franklin A.D., Amama P.B., Zakharov D.N., Stach E.A., Sands T.D., Fisher T.S. (2006). Vertical single- and double-walled carbon nanotubes grown from modified porous anodic alumina templates. Nanotechnology.

[10] Meyyappan M., Delzeit L., Cassell A., Hash D. (2003). Carbon nanotube growth by PECVD: A review. Plasma Sources Sci. Technol..

[11] Kim S.K., Lee H., Tanaka H., Weiss P.S. (2008). Vertical alignment of single-walled carbon nanotube films formed by electrophoretic deposition. Langmuir.

[12] Vijayaraghavan A., Blatt S., Weissenberger D., Oron-Carl M., Hennrich F., Gerthsen D., Hahn H., Krupke R. (2007). Ultra-large-scale directed assembly of single-walled carbon nanotube devices. Nano Lett..

[13] Goyal A., Liu S., Iqbal Z., Fetter L.A., Farrow R.C. (2008). Directed self-assembly of individual vertically aligned carbon nanotubes. J. Vac. Sci. Technol. B.

[14] Makaram P., Selvarasah S., Xiong X., Chen C.-L., Busnaina A., Khanduja N., Dokmeci M.R. (2007). Three-dimensional assembly of single-walled carbon nanotube interconnects using dielectrophoresis. Nanotechnology.

[15] Choi W.B., Jin Y.W., Kim H.Y., Lee S.J., Yun M.J., Kang J.H., Choi Y.S., Park N.S., Lee N.S., Kim J.M. (2001). Electrophoresis deposition of carbon nanotubes for triode-type field emission display. Appl. Phys. Lett..

[16] Travis J.C., Smith M.V., Rasberry S.D., Kramer G.W. (2000). Technical Specifications for Certification of Spectrophotometric NTRMs. Natl. Inst. Stand. Technol. Spec. Publ..

[17] Bai P., Li E., Lam K.T., Kurniawan O., Koh W.S. (2008). Carbon nanotube Schottky diode: An atomic perspective. Nanotechnology.

[18] Krompiewski S. (2007). Modeling a Schottky-barrier carbon nanotube field-effect transistor with ferromagnetic contacts. Nanotechnology.

[19] Franklin A.D., Farmer D.B., Haensch W. (2014). Defining and overcoming the contact resistance challenge in scaled carbon nanotube transistors. ACS Nano.

[20] Chen Z., Appenzeller J., Knoch J., Lin Y.-M., Avouris P. (2005). The role of metal−nanotube contact in the performance of carbon nanotube field-effect transistors. Nano Lett..

[21] Xiomara C.-C., Huaizhi G., Bo G., Lei A., Guohua C., Otto Z. (2009). A carbon nanotube field emission cathode with high current density and long-term stability. Nanotechnology.

[22] Bo G., Yue G., Yue Q., Cheng Y., Shimoda H., Fleming L., Zhou O. (2001). Fabrication and electron field emission properties of carbon nanotube films by electrophoretic deposition. Adv. Mater..

[23] Arnold M.S., Green A.A., Hulvat J.F., Stupp S.I., Hersam M.C. (2006). Sorting carbon nanotubes by electronic structure using density differentiation. Nat. Nanotechnol..

[24] Dresselhaus M.S., Dresselhaus G., Joriob A., Filho A.G.S., Saito R. (2002). Raman spectroscopy on isolated single wall carbon nanotubes. Carbon.

[25] Fagan J.A., Becker M.L., Chun J., Hobbie E.K. (2008). Length fractionation of carbon nanotubes using centrifugation. Adv. Mater..

[26] Fagan J.A., Lin N.J., Zeisler R., Walker A.R.H. (2011). Effects of gamma irradiation for sterilization on aqueous dispersions of length sorted carbon nanotubes. Nano Res..

[27] Kesecioglu J., Schultz M.J., Haitsma J.J., den Heetenz G.J., Lachmann B. (2002). Iodixanol inhibits exogenous surfactant therapy in rats with acute respiratory distress syndrome. Eur. Respir. J..

[28] White B., Banerjee S., O’Brien S., Turro N.J., Herman I.P. (2007). Zeta-potential measurements of surfactant-wrapped individual single-walled carbon nanotubes. J. Phys. Chem C.

[29] Boccaccini A.R., Cho J., Roether J.A., Thomas B.J.C., Minay E.J., Shaffer M.S.P. (2006). Electrophoretic deposition of carbon nanotubes. Carbon.

[30] Morgan H., Green N.G. (2003). AC Electrokinetics: Colloid and Nanoparticles.

[31] O’Brien R.W., Ward D.N. (1988). The electrophoresis of a spheroid with a thin double layer. J. Colloid Interface Sci..

[32] Hill A.V. (1910). The possible effects of the aggregation of the molecules of hæmoglobin on its dissociation curves. J. Physiol..

[33] Sze S.M., Sze S.M. (1981). Metal-semiconductor contacts. Physics of Semiconducctor Devices.

